# Fluttering in a Changing World: Effects of Urbanization and Nectar Plants on Butterfly Movement Patterns

**DOI:** 10.1002/ece3.71785

**Published:** 2025-08-11

**Authors:** Gabriela Nadine Gamrath, Moritz von der Lippe, Sascha Buchholz

**Affiliations:** ^1^ Department of Ecology Technische Universität Berlin Berlin Germany; ^2^ Berlin‐Brandenburg Institute of Advanced Biodiversity Research (BBIB) Berlin Germany; ^3^ Institute of Landscape Ecology University of Münster Münster Germany; ^4^ Centre of Integrative Biodiversity Research and Applied Ecology (CIBRA) Münster Germany

**Keywords:** behavior, *Coenonympha pamphilus*, DGPS‐tracking, *Pieris rapae*, pollinator conservation, urbanization

## Abstract

We aimed to answer the general question of whether urbanization and nectar plant abundance affect butterfly movement patterns and more specifically, (1) whether the mobility of the investigated small white ( *Pieris rapae* ) and the small heath butterfly (*Coenonympha pamphilus*) is affected differently, and (2) whether these butterflies show altered tortuosity patterns along a rural–urban gradient. The study sites were situated along a rural–urban gradient in the Berlin–Brandenburg metropolitan region (Germany). We recorded GPS movement trajectories of two common butterfly species differing in territoriality, agility, and habitat requirements, following them afoot with a DGPS backpack. Movement trajectories were analyzed in terms of mobility (flight speed and time investment in stopping, nectaring, and resting), tortuosity, and the effects of urbanization and nectar plant coverage on the derived variables were investigated using generalized linear mixed‐effect models. Nectar plant coverage negatively affected flight speed but increased flight path tortuosity for both species. *P. rapae* displayed a slightly increasing and *C. pamphilus* a slightly decreasing flight speed with increasing urbanization. With increasing urbanization, flight path tortuosity decreased for *P. rapae.* The results possibly reflect differences in the species' life history strategy, which might induce different adaptive responses in their movement behavior.

## Abstract 2

We investigated the effects of urbanization and nectar plant coverage on movement patterns of the small heath butterfly (*Coenonympha pamphilus*) and the small white butterfly ( *Pieris rapae* ). We recorded precise DGPS‐movement trajectories of the two butterfly species and analyzed them in terms of mobility and tortuosity. Generalized linear mixed‐effect models were used to illuminate the relationships between urbanization, nectar plant coverage, and the derived movement variables.

## 1 Introduction

Urbanization and its effects on wildlife represent research issues of rising importance, given the accelerating expansion of urban areas and the related decline in biodiversity (Piano et al. 2019; Simkin et al. 2022). The process of urbanization comprises biotic and abiotic alterations that, for instance, lead to changes in disturbance regimes, food quality and quantity, predation, altered competition pressure, and especially habitat fragmentation and connectivity (Seress and Liker 2015). These changes not only have the potential to weaken or efface populations, causing a decline in biodiversity or a turnover in species composition (Meckx and Van Dyck 2019; Dürrbaum et al. 2022; Szabó et al. 2023), they also may drive phenotypic or genetic alterations and (micro‐)evolutional processes in those populations that persist within cities (Alberti 2015; Merckx et al. 2018; Blattner et al. 2024). Understanding the underlying processes is essential if one aims to predict the dynamics of wildlife populations and communities in an urbanizing world and to design suitable conservation strategies to protect them (Donihue and Lambert 2014). So far, a variety of studies have found modifications in animal communication, behavior, phenology, and physiology, as well as morphological adaptations in urban environments (Alberti et al. 2017). Many of these findings are attributed to phenotypic plasticity (McDonnell and Hahs 2015), but others are proven to be hereditary (Schoville et al. 2013; Merckx et al. 2021).

Even though a great part of the studies investigating effects of urbanization on wildlife focuses on morphological or physiological attributes (Putman and Tippie 2020; Jiménez‐Peñuela et al. 2023), there is rising interest in behavioral alterations as well (Sol et al. 2013). Movement as a consequence of physical and behavioral conditions affects a variety of processes in animal life, such as foraging, escape, mate location, and reproduction (Van Dyck and Baguette 2005; Shaw 2020). It also influences important ecological processes like dispersal (Jønsson et al. 2016), and hence population connectivity, gene flow, and range shift (Shaw 2020). Movement is, therefore, one component of animal behavior with high potential for adaptive responses and thus could determine the persistence of populations facing environmental changes, like global warming and habitat fragmentation (Merckx et al. 2018; Williams and Blois 2018; Shaw 2020).

Most studies investigating the effects of urbanization on animal movement focus on mammals (Balbi et al. 2019; Li et al. 2020) or birds (Teitelbaum et al. 2020). But insects, despite their high ecological importance and potential for evolutionary adaptations in ecological time scales (sensu McDonnell and Hahs 2015), are barely represented in this specific field of research (LaPoint et al. 2015; Lookingbill et al. 2022). One possible reason for this deficit might be the high effort required to investigate insect movements, since insects are mainly too small and fragile to carry GPS devices without being affected in their behavior (Kissling et al. 2014), and it is difficult to track them visually for longer periods.

Butterflies, however, are well‐established model species for the research on movement behavior (Stevens et al. 2010). Some butterfly species are relatively easy to identify, and it is feasible to track them visually or even to follow their trajectories afoot (Fernández et al. 2016). Furthermore, the biology is well documented for many butterfly species (Cook et al. 2022), and butterflies are known to be both realistic and practical indicators for environmental change (Spaniol et al. 2020). But even though a lot of research has been conducted on butterfly movement in general (Stevens et al. 2010), and some studies compared butterfly mobility (e.g., daily movement distance) between urban and rural populations via mark‐release‐recapture procedures (Bergerot et al. 2012), there seems to be no published empirical study yet directly investigating the movement patterns of butterflies along a rural–urban gradient.

Several studies found evidence of behavioral plasticity or even heritable behavioral alterations in butterflies, caused by differing landscape structures (especially with varying degrees of habitat fragmentation), but the directionality of the observed effects seems to be inconsistent (Ducatez, Baguette, et al. 2013). This inconsistency, however, might simply reflect the different ecological requirements of the observed species (Dennis and Shreeve 1988). It appears that populations of species with rather high to intermediate sedentariness and territoriality display decreasing mobility in highly fragmented landscapes (Dennis and Shreeve 1988; Bergerot et al. 2012). Meanwhile, populations of highly vagrant, non‐territorial butterflies appear to become even more mobile when exposed to habitat fragmentation (Ducatez, Baguette, et al. 2013; Lebeau et al. 2016), possibly because populations are selected for high mobility up to a certain threshold of experienced fragmentation, above which mobility ceases to be an advantage and is selected against (Bergerot et al. 2012). Urbanization and the related changes in resource distribution (widely spaced resource patches) might also alter movement patterns in terms of smaller mean turning angles, leading to a low tortuosity of the flight paths. Since a higher linearity in movement minimizes the energy demand while maximizing the net distance moved (Scharf et al. 2009), linear and fast movement has been observed in butterflies, especially during dispersal flights in hostile environments, that is, the landscape matrix (Van Dyck and Baguette 2005). But those patterns might also arise as a considerable but weaker trend within habitats with low or scattered food resources (Scharf et al. 2009; Fernández et al. 2016).

Here we investigate two butterfly species with differing levels of sedentariness (Dennis and Shreeve 1988; Settele et al. 2005) to study the effect of urbanization and nectar plant coverage on the movement behavior of butterflies and ask if urbanization acts differently on species that vary in their territoriality and agility.

We recorded movement trajectories of *Pieris rapae* and *Coenonympha pamphilus* in terms of mobility and tortuosity along a rural–urban gradient and analyzed the data using generalized linear mixed models (GLMM). We aimed to answer the question of whether urbanization affects butterfly movement patterns and, more specifically, whether (1) the mobility of the investigated small white ( *Pieris rapae* ) and the small heath butterfly (*Coenonympha pamphilus*) is affected differently, and (2) these butterflies show altered tortuosity patterns.

## 2 Materials and Methods

### 2.1 Study Sites

**FIGURE 1:**
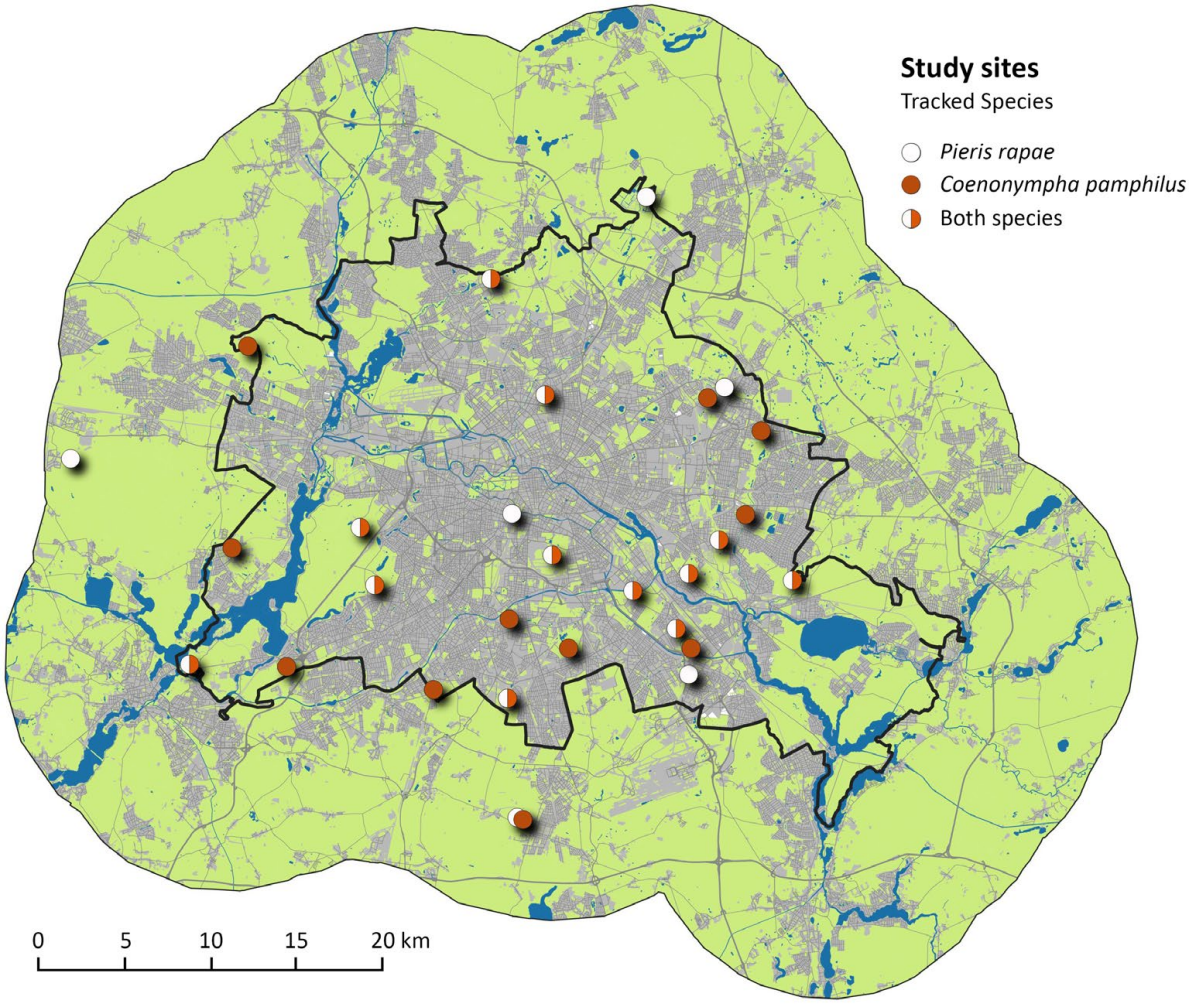
Map of the study sites (*n* = 29) within the Berlin metropolitan area (city border in black, green spaces in green, built‐up areas in gray, and water bodies in blue).

The study was conducted at 29 grassland sites in the metropolitan area of Berlin and its rural surroundings within the federal state of Brandenburg, Germany (Figure 1; 52°20′ to 52°40′ N and 12°59′ to 13°37′ E). We utilized the infrastructure of the CityScapeLabs Berlin (Von der Lippe et al. 2020), which was established to investigate urbanization effects on biodiversity and biotic interactions within the framework of the BIBS‐Project (Bridging in Biodiversity Science; https://www.tu.berlin/en/oekosys/research/projects/past‐projects). The CityScapeLab sites were preselected regarding their size (more than 1 ha), location along the rural–urban gradient (covering a wide span of urbanization), biotope type (dry or mesophilic grassland) and probable accessibility, leaving a total of 28 apparently suitable grassland sites, but only 16 of these sites were actually accessible and occupied by at least one of the investigated species. Therefore, we randomly selected and visited additional sites from the Berlin biotope type map (SenUDH 2014) and the Brandenburg biotope type map (LfU 2009), matching the same criteria, to obtain a sufficient sample size. The final study sites were dominated by herbal vegetation (herbs and grasses), but sporadically included solitary trees, shrubs, and small groves. The surroundings of the study sites ranged from virtually natural landscapes with forests and waterbodies to arable lands and detached housing to highly urbanized areas with office blocks and highways.

### 2.2 Study Species

One butterfly species that has repeatedly been studied in the context of urbanization is the small white butterfly ( *Pieris rapae* ). *P. rapae* is a generalist species that occurs in relatively high abundances, not only in (semi) natural and agricultural habitats but also in cities within and beyond its natural range (Ducatez, Humeau, et al. 2013; Kuussaari et al. 2021). This makes *P. rapae* a suitable candidate for studies on morphological (Schoville et al. 2013), genetic (Rochat et al. 2017), and behavioral (Threadgill et al. 2021) alterations in the context of anthropogenic land‐use change at different spatial scales. Another common generalist that can be found in urban areas is the small heath butterfly (*Coenonympha pamphilus*) (Gelbrecht et al. 2016). This butterfly has been classified as an indicator species of habitats with a low degree of urbanization (Bergerot et al. 2011). However, it can occur in almost any green space with herbal vegetation, as long as it is not intensely managed (Gelbrecht et al. 2016).

The small white butterfly ( *Pieris rapae* Linnaeus, 1758) and the small heath butterfly (*Coenonympha pamphilus* Linnaeus, 1758) were selected as study organisms because of their distinct degrees of territoriality and agility. While *C. pamphilus* is considered a highly territorial species of rather low mobility (Dennis and Shreeve 1988), *P. rapae* seems to be much more vagrant and rather mobile (Settele et al. 2005).

Both species are relatively abundant in the study area and have a long span of flight activity, usually lasting from March ( *P. rapae* ) or rather April (*C. pamphilus*) to October (Gelbrecht et al. 2016). Both species are nonspecific in their choice of nectar plant, but feed on specific host plants during their larval stages. *P. rapae* caterpillars mainly feed on crucifers (Brassicaceae) and *C. pamphilus* caterpillars on grasses (mainly Poaceae) (Willner 2017; Settele et al. 2005).

*P. rapae* shows evident sexual dimorphism, enabling observers to distinguish between males and females by visual cues from afar. A sex‐specific analysis of movement patterns was pursued, since butterflies show strong intersexual differences in their movement behavior. Male butterflies are known to be more mobile within habitats, while females are usually more dispersive (movement between habitats) (Reim et al. 2018). In addition, females are likely to spend a lot of time searching for suitable oviposition sites and, therefore, show different movement patterns than males (Stanton 1982).

*C. pamphilus* exhibits no apparent sexual dimorphism that can be detected from afar. Therefore, sex was not implemented in the analyses for this species.

Please note that there might have been some confusion of *P. rapae* with *Pieris mannii*, since the two species can't be distinguished on the wing and *P. mannii* was detected in Berlin for the first time in 2020 (GBIF Secretariat: GBIF Backbone Taxonomy. https://doi.org/10.15468/39omei Accessed via https://www.gbif.org/species/1920491 [2025‐01‐23]).

### 2.3 Fieldwork

The fieldwork was carried out from June to September 2020, between 9:00 and 15:00 h on sunny and calm days. To gain spatial data of butterfly movements, we tracked two to five butterflies per species per study site if the species was present ( *P. rapae* : 3.7 ± 0.9 and *C. pamphilus*: 3.4 ± 0.8 mean ± SD of butterflies per study site), by constantly keeping a distance of about 2.5 m between observer and animal. During preliminary observations, this distance had turned out to be small enough to keep the animal in sight but also large enough not to startle it by the observer's presence. Movements of the observer were logged by a DGPS receiver with real‐time differential correction (“Trimble R10,” https://help.fieldsystems.trimble.com/r10/home.htm), mounted on a GPS backpack and operated via handheld control. The GPS position was recorded with a temporal resolution of one second and an average horizontal accuracy of about 0.02 m. This method is known to be suitable for analyzing butterfly movements in different contexts (Fernández et al. 2016; Reim et al. 2018). The high accuracy of the DGPS receiver allowed for the tracking of both large‐scale fast flight as well as small‐scale fluttering flight. The openness of the landscape facilitated the initial detection and the tracking of butterflies and allowed for mostly unhindered movements of the observer and thus high conformity between butterfly and observer trajectories. Furthermore, it favored a good satellite reception and thus GPS precision. In total, movements of 138 butterflies (76 *C. pamphilus* and 62 *P. rapae* ) were analyzed. The dataset for *P. rapae* included 28 females, 34 males, and initially 5 specimens of unidentified sex, which were omitted from analyses.

In order to be able to identify stops, we noted the respective second on a prepared data sheet every time the observed butterfly was landing. Additionally, we noted the respective action of the butterfly during a stop, being either nectaring, resting, basking, or oviposition. The recording stopped when the observer lost sight of the butterfly or after a maximum of 12 min (00:05:36 ± 00:02:38; mean ± SD). All butterflies were observed by the same person, so no observer effects biased the measurements.

Since the erratic butterfly movements often made it impossible to write all information down immediately, comments regarding the tracks were recorded as audio files via “AGPTEK” Lavalier microphone on a smartphone. The audio recording started simultaneously with the GPS recording in order to achieve initial temporal synchronization. This allowed for a subsequent completion of the data sheets and facilitated the identification of stops or data gaps (the latter sometimes occurred when butterflies were flying through groves and the GPS signal was insufficient to locate the observers' position).

To obtain an approximation for food availability, a transect was drawn through the respective movement area, and the coverage of flowering nectar plants was visually estimated for each 1‐m segment of the transect within a margin of 0.5 m to the left and right sides, respectively. Only the coverage of flowering herbal vegetation and shrubs was quantified since trees were barely present within the investigated areas, and if so, eventually present tree flowers were never approached by the butterflies. The number of transect segments ranged from 4 to 82 (25.9 ± 16.0 mean ± SD) for *C. pamphilus* and from 5 to 217 (61.2 ± 44.1 mean ± SD) for *P. rapae* , depending on the net distance covered by the butterfly.

### 2.4 Data Processing

The recorded movement patterns were exported as point features in an ESRI shapefile and imported into ArcGIS 10.3.1 (Esri Inc. 2018) for processing. Line geometries were drawn along the subsequent GPS positions, using the time information (seconds since start of the track) as guidance. Due to small movements of the observer, even when trying to stand perfectly still, the butterfly landings were not recorded as perfect stops but rather as point clouds. For each point cloud, previously confirmed as a stop via comparison with the data sheet information, the median center was calculated with the ArcGIS tool “Median Center”. The computed coordinate was then attributed to all affected GPS points and used for further analyses. For the calculation of the tortuosity, however, all but the first (adjusted) point of each stop were excluded to avoid problems with the computation of the mean cosine (cf. Formula 1). Stops during initiation and termination of the GPS logging were excluded from all analyses. To describe the observer (and thereby butterfly) movements, we derived several parameters from the spatial GPS data and—referring to the two research questions—assigned them to one of the categories (1) mobility or (2) tortuosity. As measures for butterfly mobility, the mean flight speed (“flight speed” in m/s) and the share of time spent stopping (“stopping time”), nectaring (“nectaring time”), and resting (“resting time”) were calculated (Table A1, Appendix A). “Stopping time” included the time spent nectaring, resting, and pursuing other activities like basking and oviposition. Due to low sample sizes, the time spent ovipositing and the time spent basking were not analyzed separately. As a tortuosity measure, the sinuosity of the flight path, as defined by Benhamou (2004), was calculated with the R package trajr and the function TrajSinuosity2 (McLean and Skowron Volponi 2018), using the formula:

(1) $$Sin=2{\left[p\left(\frac{1+c}{1-c}+b^{2}\right)\right]}^{-0.5}$$

where *p* is the mean step length (in m), *c* is the mean cosine of turning angles, and *b* is the coefficient of variation of the step length. High values of sinuosity indicate a high tortuosity, while low values indicate a virtually straight flight path. In cases of split tracks (due to data gaps), the sinuosity was computed for each segment individually, and the mean sinuosity, weighted by relative track segment length, was then calculated for the entire butterfly track.

### 2.5 Predictors of Movement Patterns

As a measure of urbanization, the percentage of sealed soil surface around each study site was calculated. Within the boundaries of Berlin, this calculation was based on the Berlin impervious soil coverage map (BISCM, SenUDH 2016). Since such data was not available for Brandenburg, approximations based on the BISCM (SenUDH 2016) and the Berlin biotope type map (SenUDH 2014) were computed. For this purpose, the average percentage of surface sealing was calculated for each biotope type via the Zonal Statistics tool in QGIS 2.18.11 (QGIS‐DT 2018) based on the available rasterized data for Berlin (SenUDH 2016). The calculated values were then attributed to the biotope patches of the Brandenburg biotope type map (LfU 2009). The vector data gained was merged with the BISCM and then transformed into raster data (resolution: 2 × 2 m). This map was used to calculate the mean percentage of sealed surface within a 500, 1000, and 2000 m radius around the center point of each study site via the Zonal Statistics tool in QGIS 3.4.12 (QGIS‐DT 2018). These different radii were chosen because for the two investigated species, there was no data available regarding the effect radii of urbanization, but similar radii have been investigated for related species (Ducatez, Baguette, et al. 2013— *Pieris brassicae* ).

The mean nectar plant coverage of the butterfly activity range was calculated based on the field measurements using the formula:

(2) $${\mathrm{NP}}_{\mathrm{cov}}=\frac{\sum \mathrm{NP}x_{i}}{\mathrm{NTS}}$$

where $\mathrm{NP}x_{i}$ is the nectar plant coverage of the transect segment *i* (%), and NTS is the total number of transect segments. To attain an approximation for the size of the habitat patch, the area (ha) of the habitat site was calculated using the “calculate geometry” tool of ArcGIS Version 10.3.1 (Esri Inc. 2018). To do so, we first digitized the continuous grassland site (not interrupted by urban matrix, forest, agricultural crops, waterbodies or other flower‐free biotopes as reed beds) based on orthophotos of the Berlin‐Brandenburg area in summer 2020 and winter 2021 (Geoportal Berlin 2020, 2021; LGB 2020) and supported by the Berlin and Brandenburg biotope type map (LfU 2009; SenUDH 2014).

The investigated variables and random effects are also specified in Appendix A, Table A1 and the full dataset is given in Appendix B, Table A2.

### 2.6 Data Analysis

Statistical analyses were performed using the software environment R, version 3.4.3 (R Core Team 2017). We analyzed the effects of urbanization on attributes of butterfly movement using generalized linear mixed‐effect regression models (GLMM) (R package “glmmTMB”, function “glmmTMB”; Brooks et al. 2024) with habitat patch size (ha), track duration (s), and site as random covariates (intercepts). As fixed effects, the sealed soil surface (urbanization) and mean nectar plant coverage (NP coverage) were analyzed. For *P. rapae,* the factor sex was additionally included. Specimens of unidentified sex were omitted from the analyses for *P. rapae* . All numeric fixed effects were centered to their means and scaled to the respective standard deviation in order to improve comparability (function “scale” of the “base” package). The tortuosity of *C. pamphilus* was analyzed using GLMM family lognormal (link: log), while flight speed, stopping time, nectaring time, resting time, and the tortuosity of *P. rapae* were analyzed using GLMMs for beta family (link: logit), as this was the best fit according to the Cullen and Frey graph (R package “fitdistrplus,” function “descdist,” Delignette‐Muller and Dutang 2015). However, some variables needed to be transformed to fit the beta distribution. Where applicable, this is denoted in Tables 1, 2, 3. The predictor variables were previously tested for collinearity based on Spearman's pairwise correlation coefficient, with *r*s = 0.5 as the threshold value. If *r*s was ≥ 0.5, we would have excluded one of the interrelated predictors. Resulting models were analyzed regarding uniformity and dispersion of their residuals with the R package DHARMa (functions “testUniformity” and “testDispersion”; Hartig 2022). Where the residuals deviate strongly from expectations, this is denoted in Tables 1, 2, 3.

**TABLE 1:** Generalized linear mixed‐effect models (GLMM, family = beta) for mobility measures for *C. pamphilus* (*n* = 76) with regression coefficient, standard error, Wald *Z* statistic, and *p*‐value; random effects: Habitat area (ha), track duration (s), and site.

Dependent variable	Fixed effects							
	Nectar plant coverage				Urbanization			
	Estimate	Std. error	*z*	*p*	Estimate	Std. error	*z*	*p*
Flight speed^a^	**−0.085**	**0.043**	**−1.97**	0.049*	**−0.119**	**0.047**	**−2.53**	0.011*
Stopping time^b^	0.086	0.188	0.458	0.647	0.0004	0.211	0.002	0.999
Nectaring time^b^, ^c^	**0.797**	**0.256**	**3.115**	0.0018**	−0.275	0.237	−1.160	0.246
Resting time^b^, ^c^	**−0.628**	**0.201**	**−3.126**	0.0018**	−0.109	0.167	−0.653	0.514

*Note:* Residual degrees of freedom: 69. Significant relations are highlighted by bold font and asterisks.

^a^ Divided by 10 to obtain a beta‐distribution.

^b^ Small constants (1e^−10^) added to all values.

^c^ Kolmogorov–Smirnov test: Deviation significant.

**TABLE 2:** Generalized linear mixed‐effect models (GLMM, family = beta) for mobility measures for *P. rapae* (*n* = 62) with regression coefficient, standard error, Wald *Z* statistic, and *p*‐value; random effects: Habitat area (ha), track duration (s), and site.

Depend. variable	Fixed effects											
	Nectar plant coverage				Urbanization				Sex (male)			
	Estimate	Std. error	*z*	*p*	Estimate	Std. error	*z*	*p*	Estimate	Std. error	*z*	*p*
Flight speed^a^	**−0.172**	**0.050**	**−3.446**	0.001***	**0.144**	**0.058**	**2.494**	0.013*	**0.345**	**0.090**	**3.838**	< 0.001***
Stopping time^b^	**0.335**	**0.163**	**2.055**	0.040*	0.150	0.223	0.675	0.500	**−0.681**	**0.308**	**−2.210**	0.027*
Nectaring time^b^, ^c^	0.335	0.178	1.879	0.060	−0.007	0.232	−0.028	0.977	**−0.900**	**0.350**	**−2.571**	0.010*
Resting time^b^, ^c^	0.050	0.129	0.390	0.697	0.064	0.129	0.495	0.621	−0.161	0.261	−0.614	0.539

*Note:* Residual degrees of freedom: 54. Significant relations are highlighted by bold font and asterisks.

^a^ Divided by 10 to obtain a beta‐distribution.

^b^ Small constants (1e^−10^) added to all values.

^c^ KS‐test: Deviation significant.

**TABLE 3:** Generalized linear mixed‐effect models (GLMM, family = lognormal (CP) and beta (PR)) for the tortuosity of *C. pamphilus* (*n* = 76) and *P. rapae* (*n* = 62) with regression coefficient, standard error, Wald *Z* statistic, and *p*‐value; random effects: Habitat area (ha), track duration (s) and site.

Dependent variable	Fixed effects											
	Nectar plant coverage				Urbanization				Sex (male)			
	Estimate	Std. error	*z*	*p*	Estimate	Std. error	*z*	*p*	Estimate	Std. error	*z*	*p*
Tortuosity												
*C. pamphilus*	**0.076**	**0.031**	**2.449**	0.014*	0.032	0.035	0.901	0.367				
*P. rapae* ^a^	**0.182**	**0.048**	**3.785**	< 0.001***	**−0.161**	**0.064**	**−2.532**	0.011*	**−0.400**	**0.100**	**−4.012**	< 0.001***

*Note:* Residual degrees of freedom: 69 (CP) and 54 (PR). Significant relations are highlighted by bold font and asterisks.

^a^ Divided by ten to obtain beta‐distribution.

## 3 Results

Overall, *C. pamphilus* showed a lower flight speed (CP: 0.63 ± 0.24 m/s; PR: 0.98 ± 0.40 m/s; Wilcoxon rank sum test: *p* = 1.776e^−7^), higher stopping time (CP: 0.61 ± 0.28; PR: 0.43 ± 0.27; Wilcox: *p* = 6.457e^−5^), similar nectaring time (CP: 0.34 ± 0.33; PR: 0.37 ± 0.27; Wilcox: *p* = 0.347) and higher resting time (CP: 0.20 ± 0.26; PR: 0.028 ± 0.088; Wilcox: *p* = 1.481e^−7^) than *P. rapae* . Furthermore, *C. pamphilus* displayed higher flight path tortuosity (CP: 1.31 ± 0.39; PR: 0.95 ± 0.47; Wilcox: *p* = 2.717e^−7^). The mean nectar plant coverage per transect ranged from 0.3% to 42.5% (mean ± SD: 9.2% ± 8.3%), and the average urbanization around the study sites ranged from 1.4% to 68.8% (mean ± SD: 31.9% ± 17.7%).

The buffer radius at which mobility and tortuosity were best explained by urbanization was 2000 m. Thus, we only present results for the 2000 m radius regarding urbanization.

**FIGURE 2:**
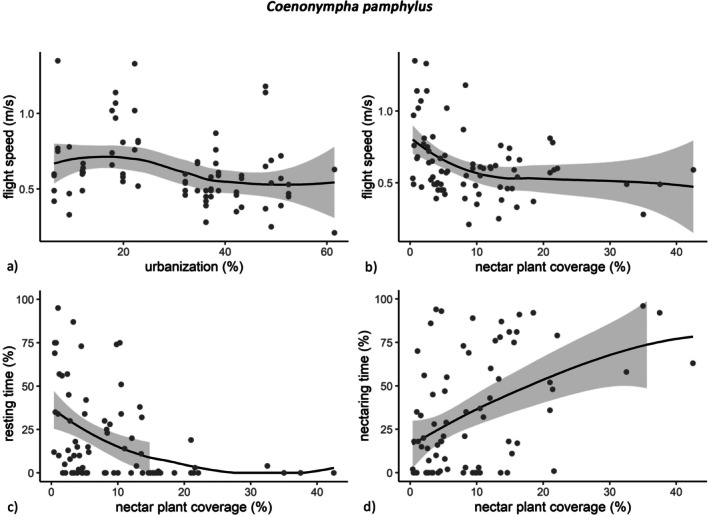
Urbanization (a) and nectar plant coverage (b, c, d) in relation to mobility measures of *Coenonympha pamphilus* (*n* = 76); with trend line (method: Loess, span: 1) and 95% confidence interval (gray ribbon); only graphs of significant relations according to GLMMs are depicted.

**FIGURE 3:**
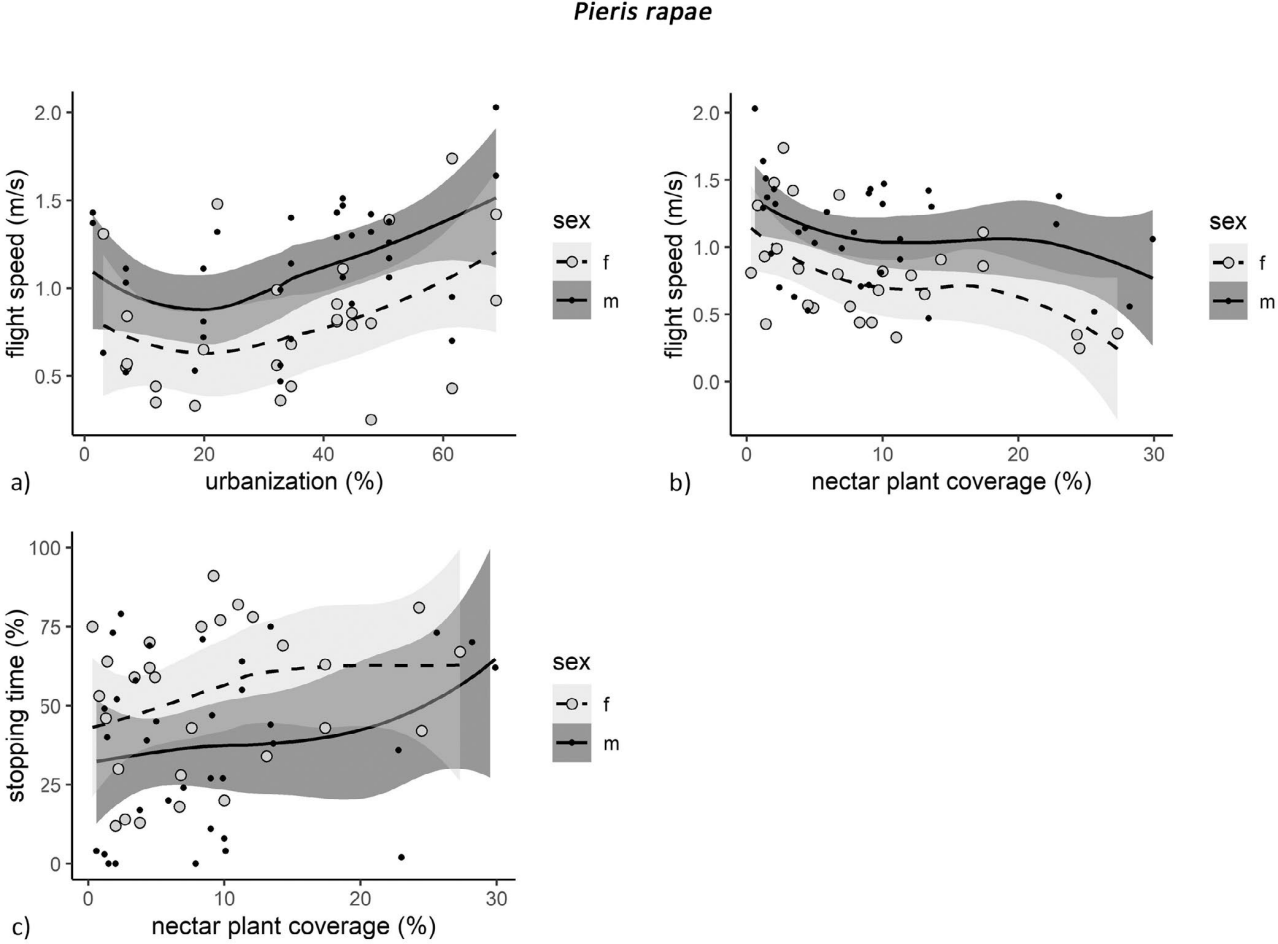
Urbanization (a) and nectar plant coverage (b, c) in relation to mobility measures of *Pieris rapae* (*n* = 62); with trend line (method: Loess, span: 1) and 95% confidence interval (gray ribbons); only graphs of significant relations according to GLMMs are depicted.

For *C. pamphilus*, urbanization slightly reduced the mean flight speed (GLMM: *coef* = −0.12, *p* = 0.011; Figure 2a; Table 1). The nectar plant coverage affected the mean flight speed in the same direction (GLMM: *coef* = −0.085, *p* = 0.049; Figure 2b; Table 1) and also reduced the resting time (GLMM: *coef* = −0.63, *p* = 0.0018; Figure 2c; Table 1), while the nectaring time was positively affected (GLMM: *coef* = 0.80, *p* = 0.0018; Figure 2d; Table 1). The flight speed of *P. rapae* was positively related to urbanization (GLMM: coef = 0.14, *p* = 0.013; Figure 3a; Table 2), but negatively related to nectar plant coverage (GLMM: coef = −0.17, *p* = 0.0006; Figure 3b; Table 2). Males had a higher flight speed than females (GLMM: coef = 0.35, *p* = 0.0001) while stopping time and nectaring time were lower for males (GLMM stopping time: coef = −0.68, *p* = 0.027; GLMM nectaring time: coef = −0.90, *p* = 0.0101; Table 2). Stopping time was positively related to nectar plant coverage in *P. rapae* (GLMM: coef = 0.34, *p* = 0.040; Figure 3c; Table 2).

**FIGURE 4:**
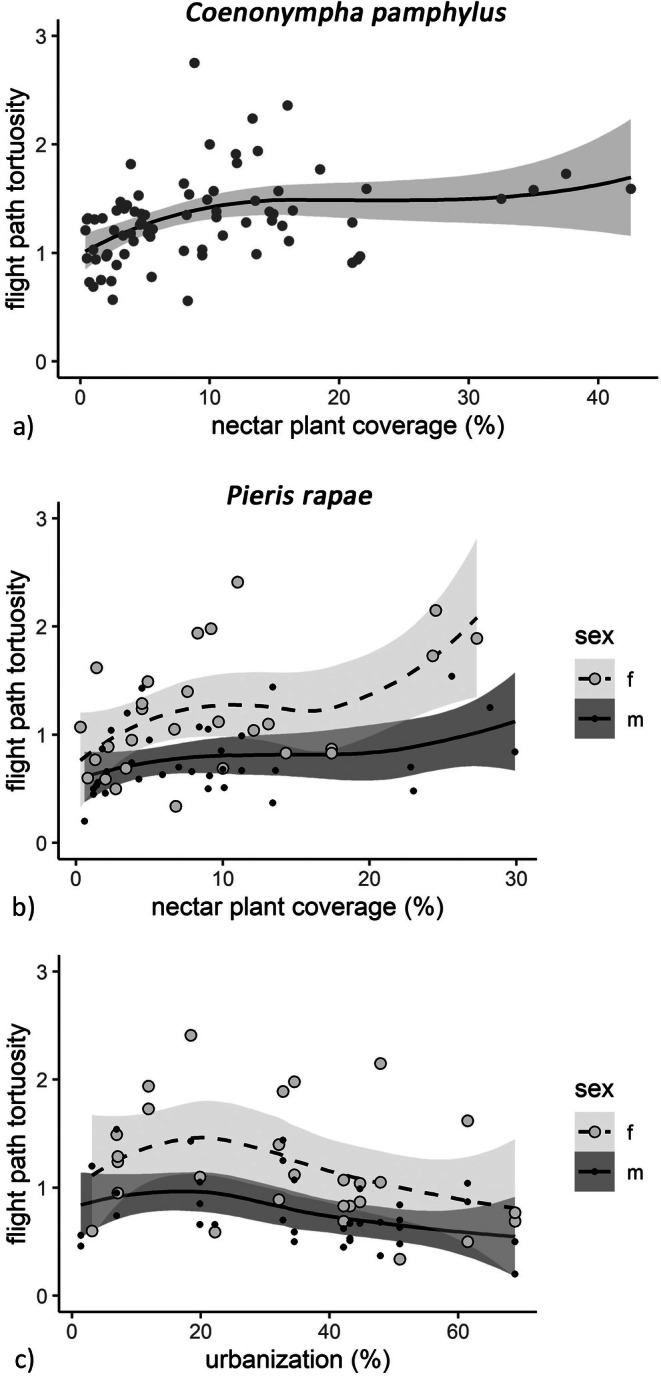
Urbanization (c) and nectar plant coverage (a, b) in relation to mean flight path tortuosity of both *C. pamphilus* (*n* = 76) and *P. rapae* (*n* = 62); with trend line (method: Loess, span: 1) and 95% confidence interval (gray ribbons); only graphs of significant relations according to GLMMs are depicted.

The flight path tortuosity was positively related to nectar plant coverage for both species (GLMM *C. pamphilus*: coef = 0.076, *p* = 0.014; GLMM *P. rapae* : coef = 0.18, *p* = 0.0002; Figure 4a,b; Table 3). Urbanization negatively affected the flight path tortuosity of *P. rapae* (GLMM: coef = −0.16, *p* = 0.011; Figure 4c; Table 3). *P. rapae* males showed an overall lower flight path tortuosity than females (GLMM: coef = −0.40, *p* < 0.001; Table 3).

Urbanization and nectar plant coverage were not considerably interrelated, neither for *C. pamphilus* nor *P. rapae* if tested for collinearity with Spearman's rank correlation (*C. pamphilus*: *r*s = 0.0256; *p* = 0.83; *P. rapae* : *r*s = 0.078; *p* = 0.53).

## 4 Discussion

### 4.1 Effects of Urbanization and Nectar Plant Coverage on the Mobility of *Coenonympha pamphilus* and *Pieris rapae*

The flight speed of *Coenonympha pamphilus* seems to be slightly negatively related to urbanization. Provided that inter‐patch movement and within‐patch movement are directly positively related in this species (see Hawkes 2009), this might be a first indicator of a stronger selection pressure toward sedentary individuals in urban environments, possibly due to the hostility of the landscape matrix and the resulting habitat fragmentation (Bergerot et al. 2012). *C. pamphilus* is a grassland‐bound species, and therefore, the habitat edge toward urban structures might act as a strong barrier. Crossing this barrier is related to costs, as the matrix might not supply sufficient nectar resources while flying is energy demanding, and the risk of predation might increase for this otherwise inconspicuous species (Merilaita et al. 2017). Thus, urbanization could fuel the selection of rather sedentary and less mobile individuals, since the costs of leaving a habitat patch outweigh the benefits of potentially finding new nectar and mate resources (Merckx et al. 2003). The recent results are in line with the findings for other butterfly species that displayed a more sedentary behavior (Schtickzelle et al. 2006: *Proclossiana eunomia*; Bergerot et al. 2012: *Pararge aegeria*) or altered flight‐related morphologies (Crawford and Keyghobadi 2018: *Lycaena epixanthe* ) in highly fragmented landscapes. However, investigations regarding the relation between the inner‐patch mobility (routine movement) and inter‐patch mobility (special movement, sensu Van Dyck and Baguette 2005) of butterflies are needed to clarify this issue. Other possible explanations for the slightly lower flight speed in urban areas are based on rather direct environmental and nonheritable effects. As mentioned above, we did not find a direct correlation between urbanization and nectar plant coverage. However, if the available nectar resources in urban habitats are limited in their amount and/or the effective sugar supply per plant due to altered habitat conditions (Gijbels et al. 2014) or through the alteration of the species composition (Williams et al. 2015), *C. pamphilus* might be urged to fly slower in order to save energy and/or to search for resources more thoroughly. Goverde et al. (2002) found that bumblebees ( *Bombus veteranus* ) visited a significantly higher percentage of flowers, flew longer distances, and spent more time within fragmented habitat patches compared with control patches. They argued that this behavioral adjustment might be the animals' strategy to optimize their energy budget in a fragmented landscape. Perhaps the observed slightly negative relation between urbanization and flight speed is a phenotypic expression of energy‐deficit compensation in *C. pamphilus*, but further investigations of the available sugar supply per plant and habitat patch would be necessary to illuminate this issue. Flight speed of *C. pamphilus* was negatively related to nectar plant coverage, probably reflecting intensive local searching behavior when potential nectar resources are abundant. For example, Evans et al. (2020) found slow and tortuous flight in the meadow brown butterfly (*Maniola jurtina*) to be associated with nectaring behavior. Consistently, Fernández et al. (2016) recorded slower movements in the silver‐studded blue butterfly (*Plebejus argus*) in high‐quality than low‐quality habitats. Contrary to expectations, neither the overall time investment in stopping nor in nectaring or resting of *C. pamphilus* was related to urbanization. Provided that urbanization lowers the energy supply within habitat patches, it might, however, not affect resting or nectaring behavior if the experienced energy deficit is sufficiently compensated by a lower flight speed (Abrol 2005). Since take‐off flight is especially energy demanding (Vande Velde and Van Dyck 2013), an increased stop (and thus take‐off) frequency could be ineffective for balancing energy deficits. Prolonged resting bouts might lower the energy demand, but the energy intake as well.

The nectaring time of *C. pamphilus* increased with nectar plant coverage, while the opposite was true for the resting time. This might simply reflect the utilization of present resources and the related trade‐off between the time investment in resting and foraging (Szigeti et al. 2018). However, the KS‐test revealed significant deviation of the models' residues from the expected distribution and thus a nonoptimal model fit. The results for nectaring time and resting time should, therefore, be interpreted with reservations.

As expected, the mean flight speed of *P. rapae* was positively related to urbanization, while there was an even stronger but negative relation to nectar plant coverage. This might indicate that a main determinant of flight speed in *P. rapae* is resource availability, with high nectar plant coverage resulting in a more local and slower search behavior, while low nectar plant coverage leads to directional and fast flight. These results are in line with findings of other studies investigating butterfly movements in relation to resource availability (Fernández et al. 2016; Evans et al. 2020). However, the current resource availability does not seem to be the only driving factor of *P. rapae* mobility. Urbanization might act as a driver, selecting for mobile individuals who are able to cross the hostile environment quickly. Provided that inter‐patch mobility and inner‐patch mobility are positively related in *P. rapae* (see Thomas 2000; Van Dyck and Baguette 2005; Hawkes 2009), this selection would also affect the measured flight speed within habitat patches. Consequently, Schoville et al. (2013) found morphological variations in the wing size and genetic traits of *P. rapae* along a rural–urban gradient, with males having larger wings in urban than rural populations. They linked the larger wing size to a higher dispersal ability (also see Sekar 2012). Lebeau et al. (2016) found a higher flight endurance in butterflies (*M. jurtina*) from sites with intensive agriculture than extensive agriculture. This could be attributed to the fact that in landscapes with highly scattered resource or habitat patches, the butterfly populations of mobile species are selected for individuals with better‐developed thoracic musculature that, in turn, leads to increased flight performance and thus greater mobility (Thomas et al. 1998).

Overall, *P. rapae* males displayed a higher flight speed and lower stopping time and nectaring time than females. Better movement capacities of males compared with females were found in several butterfly species (Bakowski et al. 2010; Reim et al. 2018), including *P. rapae* (Ducatez, Humeau, et al. 2013). This is often explained by different selection pressures acting on males and females (Ducatez, Humeau, et al. 2013). It has repeatedly been stated that female butterflies experience a trade‐off between mobility and fecundity (Gibbs and Van Dyck 2010). Thus, the fitness rewards due to increased mobility might be lower for females than for males, if the former experience higher mobility only at the expense of fecundity. However, the concept of the mobility–fecundity trade‐off has been challenged by some authors. Hanski et al. (2006) found higher lifetime fecundity in more mobile females of the Glanville fritillary butterfly (*Melitaea cinxia*) than in less mobile ones. The trade‐off might depend on food availability and species‐specific life‐history traits. Thus, this issue is in need of further investigation.

As Bergerot et al. (2012) argued, within an increasingly fragmented landscape, selection usually promotes mobile individuals until a certain threshold of fragmentation is reached. When this threshold is exceeded, the species' mobility is likely to experience a reversed selection toward sedentariness (Baguette and Schtickzelle 2006). Since the level of effective habitat fragmentation not only depends on the spatial configuration and quality of habitat and matrix, but also the focal species itself (Stevens et al. 2010), it is not surprising that the two investigated species seem to develop contrasting mobility trends in the context of urbanization. *C. pamphilus* and *P. rapae* differ markedly in a variety of traits, including their territoriality, agility, and habitat requirements (Settele et al. 2005). These properties are often interrelated, making it difficult to pinpoint the main driver of phenotypic or genotypic differentiations (Ewers and Didham 2006). Whether our findings actually indicate evolutionary adaptation, phenotypic plasticity, parental or environmental effects (see Shaw 2020; Braem and Van Dyck 2023) remains unclear and could be the subject of further investigations including common garden experiments on uniformly reared filial generations and split‐brood experiments (sensu Meckx and Van Dyck 2006; Donihue and Lambert 2014).

Since multiple studies found a strong trade‐off between mobility and fecundity in butterflies (Gibbs et al. 2010; Duplouy et al. 2013), the question arises whether a potential trend toward higher mobility in *P. rapae* (and possibly other species, experiencing similar pressures)—even though apparently advantageous—may have negative long‐term effects on reproductive success and the persistence of urban populations. On the other hand, a trend toward more sedentary populations in urban areas, as indicated for *C. pamphilus* (especially if this trend was genetically fixed), would be even more concerning. In such highly dynamic environments, strong sedentariness could lead to local extinctions if animals are not able to reach suitable habitat patches when their initial habitat is destroyed (e.g., through building activities) or becomes too small to maintain a population (Thomas and Hanski 1997). Furthermore, a decrease in genetic exchange between urban subpopulations may lead to inbreeding effects and thus potentially to reduced fitness (Rochat et al. 2017). Indeed, multiple studies analyzing butterfly communities found species with low and intermediate mobility to be more sensitive to urbanization and habitat fragmentation than mobile species (Öckinger et al. 2010; Kuussaari et al. 2021).

### 4.2 Effects of Urbanization and Nectar Plant Coverage on Flight Path Tortuosity of *Coenonympha pamphilus* and *Pieris rapae*

The flight path tortuosity of *P. rapae* was negatively related to urbanization, indicating a tendency toward straighter movement patterns in urban environments. *P. rapae* , as a mobile and non‐territorial species, might benefit from directed straight flight when resources are scattered and patchily distributed, which is often the case in urban environments (Kuussaari et al. 2021). The flight path tortuosity of *C. pamphilus*, however, showed a nonsignificant and weakly positive relation to urbanization. This lack of a linear relation possibly occurs because *C. pamphilus*, as a rather immobile and territorial species, experiences urban habitats as strongly isolated, and the available resources are aggregated in a small spatial extent. Thus, *C. pamphilus* might not benefit from straighter flight, as a big part of the population presumably is not able to cross the urban matrix and reach distant habitat patches, but maximizes its fitness by staying within the suitable habitat.

The tortuosity of both study species was positively related to nectar plant coverage, indicating an intensified local search behavior when food resources are abundant (Fernández et al. 2016; Evans et al. 2020). Male *P. rapae* had an overall lower flight path tortuosity than females, which is in line with the results of other studies regarding sexual differences in movement tortuosity of butterflies and might be attributed to the frequent male attempt to examine large areas in order to find receptive females (Skórka et al. 2013) and/or the high tortuosity in females during host plant search.

Overall, we found noteworthy relations between urbanization, nectar plant coverage, and flight speed in *C. pamphilus* and *P. rapae* , with opposing effects of urbanization on the flight speed of the two investigated species. The tortuosity of both species was positively related to nectar plant coverage, but only the tortuosity of *P. rapae* was negatively related to urbanization. The differences between the effects of urbanization on the mobility and tortuosity of these species can most likely be attributed to their differing life history strategies, but this issue is in need of further research, especially regarding the inheritance and generalizability of the observed trends. Our results show that not only the resource availability within a habitat patch but also the surrounding degree of urbanization can affect the movement patterns of butterflies in urban areas.

### 4.3 Methodological Limitations

We want to emphasize that even though the observer followed butterflies with the highest accuracy possible, a human being does of course experience different physical limitations than a butterfly, and therefore some inaccuracies in speed and flight path might have occurred, especially when obstacles (e.g., fences or small shrubs) temporarily slowed down or hindered the observer but not the butterfly. We, however, are confident that the recordings realistically reflect the overall flight behavior of the investigated butterflies, but encourage further methodological refinement in this field of research. We also want to stress that further studies with higher sample sizes (more butterflies per study site and more study sites), as well as repeating the study in different cities and for different species, will be beneficial to check the validity and repeatability of the observed (partly weak) trends. With respect to the growing distribution area of *P. mannii* , future studies on *P. rapae* should consider its potential coexistence with and its distinction from *P. mannii* .

## Author Contributions

**Gabriela Nadine Gamrath:** data curation (lead), formal analysis (lead), investigation (lead), methodology (equal), visualization (lead), writing – original draft (equal), writing – review and editing (equal). **Moritz von der Lippe:** conceptualization (equal), methodology (equal), supervision (equal), visualization (supporting), writing – review and editing (equal). **Sascha Buchholz:** conceptualization (equal), methodology (equal), supervision (equal), validation (equal), writing – original draft (equal), writing – review and editing (equal).

## Conflicts of Interest

The authors declare no conflicts of interest.

## Data Availability

Data are available from the Dryad Digital Repository (https://doi.org/10.5061/dryad.bk3j9kdnn).

## Appendix A

**TABLE A1:** Investigated variables and random effects, with unit or range, formula, method, equipment, software, data sources, and/or references used to calculate these measures.

	Variable	Unit/range	Formula		Method/equipment/software	Data sources/references
Independent variables						
Site specific	Urbanization	%	$\text{Sealing}=\frac{\sum \text{Seal}x_{i}}{\mathrm{Pix}}$	Seal*x*_*i*_ = sealed surface of 2 × 2 m pixel *i* (%) Pix = total number of pixels within the 2000 m buffer radius Sealing = sealed surface within 2000 m buffer radius (%)	Software: QGIS Version 3.4.12 (QGIS‐DT 2018), Tool: Zonal Statistics	Berlin Environmental Atlas/Impervious Soil Coverage Map (SenUDH 2016), Berlin Biotope Type Map (SenUDH 2014), Brandenburg Biotope Type Map (LfU 2009)
	Habitat size	ha			Software: ArcGIS Version 10.3.1 (Esri Inc. 2018), Tool: calculate geometry	Orthophotos of the Berlin‐Brandenburg area in summer 2020 and winter 2021 (Geoportal Berlin 2020, 2021; LGB 2020) and the Berlin and Brandenburg biotope type map (LfU 2009, SenUDH 2014)
Track specific	Track duration	s			GPS Unit “Trimble R10,” Software: ArcGIS Version 10.3.1 (Esri Inc. 2018), Microsoft Excel 2007 (Microsoft Corporation 2007)	
	Nectar plant coverage	%	${\mathrm{NP}}_{\mathrm{cov}}=\frac{\sum \mathrm{NP}x_{i}}{\mathrm{NTS}}$	NP*x*_*i*_ = nectar plant coverage of transect segment *i* (%) NTS = number of transect segments	Visual estimation, GPS Unit “Trimble R10,” Software: ArcGIS Version 10.3.1 (Esri Inc. 2018), Microsoft Excel 2007 (Microsoft Corporation 2007)	
	Sex	Female/male/NA			Visual classification in the field	
Dependent variables						
Mobility	Flight speed	m/s	$\mathrm{vF}=\frac{L}{\left(\mathrm{dur}T-\sum \;\mathrm{dur}S_{i}\right)}$	*L* = length of the flight path (m) dur*T* = duration of track (s)^a^ dur*S*_*i*_ = duration of stop *i* (s)^a^	GPS Unit “Trimble R10,” Software: ArcGIS Version 10.3.1 (Esri Inc. 2018), Microsoft Excel 2007 (Microsoft Corporation 2007)	
	Stopping time	0–1	$\mathrm{ShS}=\frac{\sum \;\mathrm{dur}S_{i}}{\mathrm{dur}T}$	dur*S*_*i*_ = duration of stop *i* (s)^a^ dur*T* = duration of track (s)^a^	Same as above	
	Nectaring time	0–1	$ShN=\frac{\sum \;\mathrm{dur}N_{i}}{\mathrm{dur}T}$	dur*N*_*i*_ = duration of nectaring bout *i* (s)^a^ dur*T* = duration of track (s)^a^	Same as above	
	Resting time	0–1	$\mathrm{ShR}=\frac{\sum \;\mathrm{dur}R_{i}}{\mathrm{dur}T}$	dur*R*_*i*_ = duration of resting bout *i* (s)^a^ dur*T* = duration of track (s)^a^	Same as above	
Tortuosity	Sinuosity	Nondimensional	$\mathrm{Sin}=2{\left[p\left(\frac{1+c}{1-c}+b^{2}\right)\right]}^{-0.5}$	*p* = mean step length (m) *c* = mean cosine of turning angles *b* = coefficient of variation of the step length	GPS Unit “Trimble R10,” Software: ArcGIS 10.3.1 Version (Esri Inc. 2018), Microsoft Excel 2007 (Microsoft Corporation 2007), RStudio (RStudio Inc. 2018) Package: trajr, Function: TrajSinuosity2 (McLean and Skowron Volponi 2018)	Benhamou (2004)

^a^ Stops during initiation and termination of GPS‐logging were excluded from the analyses.

## Appendix B

**TABLE A2:** Dataset with variables included in the statistical analyses.

Site	BID	Spec	Sex	Habitat size (ha)	Track duration (s)	Urbanization (%)	NP coverage (%)	Flight speed (m/s)	Stopping time	Nectaring time	Resting time	Sinuosity
ABBB	ABBB_01	CP	NA	5.37	400.00	9.34	21.38	0.78	0.48	0.48	0.00	0.94
	ABBB_02	CP	NA	5.37	390.00	9.34	13.50	0.47	0.89	0.78	0.11	1.48
	ABBB_03	CP	NA	5.37	495.00	9.34	16.00	0.33	0.81	0.81	0.00	2.36
AGNK	AGNK_01	PR	f	16.79	389.00	44.72	12.06	0.79	0.78	0.05	0.12	1.04
	AGNK_02	PR	f	16.79	310.00	44.72	17.41	0.86	0.63	0.63	0.00	0.87
	AGNK_03	PR	m	16.79	491.00	44.72	13.63	1.30	0.38	0.38	0.00	0.67
	AGNK_04	PR	m	16.79	250.00	44.72	11.26	0.91	0.64	0.64	0.00	0.99
BGBR	BGBR_01	CP	NA	4.04	666.00	36.21	35.00	0.28	0.96	0.96	0.00	1.58
	BGBR_02	CP	NA	4.04	480.00	36.21	32.48	0.49	0.61	0.58	0.04	1.50
	BGBR_04	CP	NA	4.04	436.00	36.21	10.31	0.42	0.78	0.03	0.75	1.57
	BGBR_06	CP	NA	4.04	423.00	36.21	3.88	0.39	0.94	0.94	0.00	1.82
	BGBR_07	CP	NA	4.04	471.00	36.21	5.00	0.45	0.55	0.21	0.34	1.35
BMBB	BMBB_02	PR	m	2.95	495.00	6.91	25.59	0.52	0.73	0.73	0.00	1.54
	BMBB_03	PR	m	2.95	325.00	6.91	5.00	1.03	0.45	0.45	0.00	0.95
	BMBB_04	PR	f	2.95	79.00	6.91	4.92	0.55	0.59	0.59	0.00	1.49
	BMBB_06	PR	m	2.95	166.00	6.91	3.82	1.11	0.17	0.17	0.00	0.74
BSLB	BSLB_01	CP	NA	37.43	408.00	34.52	1.03	0.67	0.69	0.35	0.34	1.03
	BSLB_02	CP	NA	37.43	519.00	34.52	0.52	0.49	0.84	0.00	0.75	1.31
	BSLB_03	CP	NA	37.43	420.00	34.52	1.14	0.68	0.80	0.70	0.10	1.31
	BSLB_04	PR	f	37.43	691.00	34.52	9.20	0.44	0.91	0.91	0.00	1.98
	BSLB_05	PR	m	37.43	220.00	34.52	4.31	1.14	0.39	0.00	0.39	0.59
	BSLB_06	PR	m	37.43	160.00	34.52	9.02	1.40	0.11	0.11	0.00	0.50
	BSLB_07	PR	f	37.43	532.00	34.52	9.73	0.68	0.77	0.63	0.02	1.12
	BSLB_08	PR	m	37.43	408.00	34.52	8.39	0.71	0.71	0.71	0.00	1.07
CIMM	CIMM_01	CP	NA	27.20	269.00	38.65	42.50	0.59	0.65	0.63	0.00	1.59
	CIMM_02	CP	NA	27.20	336.00	38.65	12.00	0.61	0.63	0.43	0.00	1.91
	CIMM_03	CP	NA	27.20	567.00	38.65	37.50	0.49	0.92	0.92	0.00	1.73
CSSA	CSSA_01	CP	NA	1.01	454.00	49.02	5.21	0.69	0.61	0.08	0.42	1.18
	CSSA_02	CP	NA	1.01	738.00	49.02	3.43	0.54	0.20	0.00	0.00	1.41
	CSSA_03	CP	NA	1.01	556.00	49.02	13.29	0.25	0.92	0.54	0.38	2.24
DFSZ	DFSZ_01	CP	NA	1.19	424.00	17.73	14.93	0.74	0.81	0.81	0.00	1.36
	DFSZ_02	CP	NA	1.19	381.00	17.73	16.43	0.66	0.91	0.91	0.00	1.39
	DFSZ_03	CP	NA	1.19	454.00	17.73	5.51	1.02	0.70	0.55	0.15	0.78
DHBB	DHBB_01	PR	m	641.03	140.00	3.10	3.47	0.63	0.58	0.58	0.00	1.20
	DHBB_02	PR	f	641.03	462.00	3.10	0.80	1.31	0.53	0.53	0.00	0.60
	DHBB_03	PR	NA	641.03	402.00	3.10	0.33	1.11	0.18	0.18	0.00	0.60
DHGO	DHGO_01	PR	m	10.61	55.00	19.88	7.90	1.11	0.00	0.00	0.00	0.66
	DHGO_02	PR	f	10.61	50.00	19.88	13.10	0.65	0.34	0.34	0.00	1.10
	DHGO_03	CP	NA	10.61	342.00	19.88	8.40	0.55	0.58	0.35	0.23	1.54
	DHGO_04	PR	m	10.61	308.00	19.88	9.88	0.81	0.27	0.27	0.00	0.85
	DHGO_05	CP	NA	10.61	121.00	19.88	22.14	0.60	0.79	0.79	0.00	1.59
	DHGO_06	CP	NA	10.61	127.00	19.88	20.97	0.81	0.52	0.52	0.00	0.91
	DHGO_07	PR	m	10.61	490.00	19.88	9.00	0.72	0.27	0.27	0.00	1.05
	DHGO_08	CP	NA	10.61	119.00	19.88	4.71	0.58	0.93	0.93	0.00	1.36
DNDP	DNDP_01	PR	m	3.01	466.00	61.45	2.37	0.70	0.79	0.56	0.05	1.04
	DNDP_02	PR	NA	3.01	131.00	61.45	4.86	0.74	0.44	0.30	0.14	1.04
	DNDP_03	PR	f	3.01	76.00	61.45	1.38	0.43	0.64	0.64	0.00	1.62
	DNDP_04	CP	NA	3.01	512.00	61.45	8.75	0.21	0.97	0.69	0.28	2.75
	DNDP_05	PR	m	3.01	510.00	61.45	1.75	0.95	0.73	0.26	0.00	0.87
	DNDP_06	CP	NA	3.01	115.00	61.45	9.38	0.63	0.83	0.00	0.00	1.03
	DNDP_07	PR	f	3.01	84.00	61.45	2.69	1.74	0.14	0.14	0.00	0.50
FJTK	FJTK_01	PR	f	68.72	325.00	47.91	24.53	0.25	0.42	0.08	0.31	2.15
	FJTK_02	PR	m	68.72	208.00	47.91	13.44	1.42	0.75	0.02	0.00	0.37
	FJTK_03	PR	f	68.72	121.00	47.91	6.65	0.80	0.18	0.14	0.04	1.05
FJTK	FJTK_04	CP	NA	68.72	80.00	47.91	8.34	1.18	0.28	0.03	0.25	0.56
	FJTK_05	PR	m	68.72	165.00	47.91	10.00	1.32	0.08	0.08	0.00	0.68
	FJTK_06	CP	NA	68.72	373.00	47.91	3.30	0.65	0.87	0.00	0.87	1.16
	FJTK_07	CP	NA	68.72	561.00	47.91	2.55	1.14	0.88	0.00	0.13	0.57
	FJTK_08	CP	NA	68.72	403.00	47.91	18.50	0.37	0.92	0.92	0.00	1.77
FPMF	FPMF_01	CP	NA	12.11	311.00	32.13	3.58	0.49	0.45	0.28	0.18	1.44
	FPMF_02	CP	NA	12.11	498.00	32.13	10.53	0.55	0.88	0.37	0.51	1.38
	FPMF_03	CP	NA	12.11	412.00	32.13	9.40	0.60	0.89	0.89	0.00	0.98
	FPMF_04	PR	f	12.11	283.00	32.13	7.58	0.56	0.43	0.42	0.02	1.40
	FPMF_05	CP	NA	12.11	93.00	32.13	14.64	0.46	0.00	0.00	0.00	1.38
	FPMF_06	CP	NA	12.11	474.00	32.13	16.08	0.54	0.18	0.17	0.01	1.11
	FPMF_07	PR	f	12.11	105.00	32.13	2.15	0.99	0.30	0.07	0.24	0.89
GAHS	GAHS_01	CP	NA	79.67	190.00	12.04	10.50	0.61	0.34	0.00	0.34	1.33
	GAHS_02	CP	NA	79.67	384.00	12.04	2.84	0.64	0.76	0.00	0.30	1.39
	GAHS_03	CP	NA	79.67	419.00	12.04	4.47	0.67	0.74	0.01	0.73	1.53
GPFK	GPFK_02	PR	f	5.54	314.00	68.82	3.43	1.42	0.59	0.59	0.00	0.69
	GPFK_03	PR	m	5.54	78.00	68.82	1.23	1.64	0.03	0.03	0.00	0.50
	GPFK_05	PR	m	5.54	70.00	68.82	0.64	2.03	0.04	0.04	0.00	0.20
	GPFK_06	PR	f	5.54	420.00	68.82	1.31	0.93	0.46	0.46	0.00	0.77
GWMH	GWMH_01	CP	NA	9.70	287.00	38.15	13.63	0.76	0.56	0.00	0.32	0.99
	GWMH_02	CP	NA	9.70	509.00	38.15	7.95	0.87	0.72	0.00	0.30	1.02
	GWMH_03	CP	NA	9.70	430.00	38.15	14.80	0.67	0.68	0.18	0.00	1.30
KHTK	KHTK_01	PR	m	1.25	95.00	43.19	1.35	1.51	0.40	0.40	0.00	0.53
	KHTK_03	PR	m	1.25	52.00	43.19	10.07	1.47	0.04	0.04	0.00	0.51
	KHTK_04	PR	f	1.25	120.00	43.19	17.44	1.11	0.43	0.43	0.00	0.83
	KHTK_05	PR	m	1.25	244.00	43.19	11.31	1.06	0.55	0.08	0.47	0.67
	KHTK_06	CP	NA	1.25	406.00	43.19	13.70	0.38	0.87	0.87	0.00	1.94
	KHTK_07	CP	NA	1.25	466.00	43.19	15.60	0.59	0.75	0.75	0.00	1.25
	KHTK_08	CP	NA	1.25	341.00	43.19	21.00	0.57	0.56	0.36	0.19	1.28
LFSZ	LFSZ_01	CP	NA	40.82	458.00	22.86	3.43	0.82	0.55	0.45	0.10	0.99
	LFSZ_02	CP	NA	40.82	475.00	22.86	2.12	0.81	0.56	0.56	0.00	0.99
	LFSZ_03	CP	NA	40.82	388.00	22.86	3.08	0.52	0.86	0.86	0.00	1.47
MHFK	MHFK_01	CP	NA	6.95	126.00	22.19	2.44	1.33	0.57	0.00	0.57	0.74
	MHFK_02	CP	NA	6.95	336.00	22.19	1.17	1.02	0.75	0.18	0.57	0.94
	MHFK_03	CP	NA	6.95	390.00	22.19	0.61	0.76	0.53	0.18	0.35	1.32
	MHFK_04	PR	m	6.95	321.00	22.19	2.11	1.32	0.52	0.52	0.00	0.66
	MHFK_05	PR	f	6.95	276.00	22.19	2.02	1.48	0.12	0.12	0.00	0.59
	MHFK_06	PR	NA	6.95	510.00	22.19	2.56	1.45	0.05	0.05	0.00	0.47
NSGM	NSGM_04	CP	NA	2.82	476.00	52.46	4.67	0.45	0.21	0.18	0.03	1.28
	NSGM_05	CP	NA	2.82	518.00	52.46	0.40	0.53	0.16	0.02	0.12	1.21
	NSGM_06	CP	NA	2.82	422.00	52.46	1.73	0.47	0.15	0.15	0.00	1.32
NWMM	NWMM_01	PR	m	22.60	510.00	32.77	28.24	0.56	0.70	0.70	0.00	1.25
	NWMM_02	PR	m	22.60	293.00	32.77	13.39	0.47	0.44	0.43	0.02	1.44
	NWMM_03	PR	m	22.60	480.00	32.77	7.02	0.99	0.24	0.24	0.00	0.70
	NWMM_04	PR	f	22.60	480.00	32.77	27.33	0.36	0.67	0.67	0.00	1.89
RFHF	RFHF_01	PR	m	138.87	88.00	1.36	2.04	1.43	0.00	0.00	0.00	0.46
	RFHF_02	PR	m	138.87	176.00	1.36	1.53	1.37	0.00	0.00	0.00	0.56
	RFHF_03	PR	NA	138.87	102.00	1.36	1.88	1.49	0.00	0.00	0.00	0.49
SFWE	SFWE_01	CP	NA	47.71	270.00	6.37	11.02	0.60	0.46	0.32	0.14	1.16
	SFWE_02	CP	NA	47.71	72.00	6.37	5.16	0.42	0.47	0.47	0.00	1.24
	SFWE_03	CP	NA	47.71	336.00	6.37	4.55	0.49	0.18	0.18	0.00	1.26
	SFWE_06	CP	NA	47.71	535.00	6.37	21.61	0.59	0.04	0.01	0.03	0.97
SGSZ	SGSZ_01	PR	f	7.77	215.00	7.08	3.77	0.84	0.13	0.02	0.01	0.95
	SGSZ_02	PR	f	7.77	493.00	7.08	4.50	0.57	0.62	0.62	0.00	1.24
	SGSZ_03	CP	NA	7.77	95.00	7.08	0.67	1.35	0.75	0.00	0.75	0.73
	SGSZ_04	PR	f	7.77	498.00	7.08	4.52	0.57	0.70	0.70	0.00	1.29
	SGSZ_05	CP	NA	7.77	438.00	7.08	1.96	0.77	0.25	0.20	0.05	0.97
	SGSZ_06	CP	NA	7.77	458.00	7.08	2.56	0.75	0.59	0.14	0.45	1.21
SIJG	SIJG_01	PR	f	3.01	219.00	11.89	8.33	0.44	0.75	0.75	0.00	1.94
	SIJG_02	PR	f	3.01	440.00	11.89	24.33	0.35	0.81	0.81	0.00	1.73
	SIJG_04	CP	NA	3.01	178.00	11.89	12.14	0.60	0.80	0.60	0.20	1.83
	SIJG_05	CP	NA	3.01	318.00	11.89	8.00	0.49	0.73	0.73	0.00	1.64
	SIJG_06	PR	NA	3.01	381.00	11.89	15.93	0.48	0.77	0.77	0.00	1.45
	SIJG_07	CP	NA	3.01	465.00	11.89	12.83	0.62	0.80	0.76	0.04	1.28
SWMH	SWMH_02	CP	NA	8.11	508.00	37.18	5.56	0.58	0.14	0.02	0.12	1.22
	SWMH_03	CP	NA	8.11	220.00	37.18	4.24	0.45	0.10	0.00	0.10	1.38
	SWMH_04	CP	NA	8.11	519.00	37.18	4.06	0.50	0.19	0.16	0.02	1.11
	SWMH_05	CP	NA	8.11	508.00	37.18	3.91	0.49	0.27	0.10	0.15	1.18
TFLT	TFLT_01	CP	NA	70.91	350.00	18.44	1.00	1.14	0.95	0.00	0.95	0.69
	TFLT_02	CP	NA	70.91	444.00	18.44	0.48	0.97	0.69	0.00	0.69	0.95
	TFLT_03	PR	f	70.91	304.00	18.44	11.00	0.33	0.82	0.82	0.00	2.41
	TFLT_04	PR	m	70.91	349.00	18.44	4.55	0.53	0.69	0.69	0.00	1.43
	TFLT_05	CP	NA	70.91	376.00	18.44	1.63	1.07	0.90	0.33	0.56	0.75
THFT	THFT_01	CP	NA	60.66	496.00	50.93	8.16	0.39	0.21	0.21	0.00	1.35
	THFT_02	CP	NA	60.66	300.00	50.93	2.78	0.72	0.16	0.07	0.08	0.89
	THFT_03	CP	NA	60.66	237.00	50.93	5.36	0.57	0.29	0.29	0.00	1.15
	THFT_04	PR	f	60.66	289.00	50.93	6.79	1.39	0.28	0.28	0.00	0.34
	THFT_05	PR	m	60.66	331.00	50.93	29.86	1.06	0.62	0.60	0.02	0.84
	THFT_06	PR	m	60.66	498.00	50.93	22.76	1.17	0.36	0.25	0.00	0.70
	THFT_07	PR	m	60.66	186.00	50.93	23.00	1.38	0.02	0.02	0.00	0.48
	THFT_08	PR	m	60.66	399.00	50.93	5.90	1.26	0.20	0.20	0.00	0.63
WHTK	WHTK_01	PR	m	15.76	88.00	42.20	1.18	1.29	0.49	0.49	0.00	0.45
	WHTK_02	PR	f	15.76	254.00	42.20	0.25	0.81	0.75	0.75	0.00	1.07
	WHTK_03	PR	m	15.76	328.00	42.20	9.13	1.43	0.47	0.47	0.00	0.62
	WHTK_04	PR	f	15.76	140.00	42.20	14.27	0.91	0.69	0.69	0.00	0.83
	WHTK_06	PR	f	15.76	267.00	42.20	9.99	0.82	0.20	0.16	0.04	0.69
	WHTK_07	CP	NA	15.76	484.00	42.20	15.31	0.46	0.93	0.11	0.00	1.57
	WHTK_08	CP	NA	15.76	144.00	42.20	9.81	0.48	0.74	0.00	0.74	1.49
	WHTK_09	CP	NA	15.76	285.00	42.20	10.00	0.35	0.96	0.00	0.00	2.00

Abbreviations: BID, butterfly identifier; NP, nectar plant; Site, study site; Spec, species (CP—*Coenonympha pamphylus*, PR – *Pieris rapae* ).
